# Intimate Partner Violence: A Bibliometric Review of Literature

**DOI:** 10.3390/ijerph17155607

**Published:** 2020-08-04

**Authors:** Yanqi Wu, Jie Chen, Hui Fang, Yuehua Wan

**Affiliations:** 1 Institute of Information Resource, Zhejiang University of Technology, Hangzhou 310014, China; wuyanqi@zjut.edu.cn (Y.W.); chjie@zjut.edu.cn (J.C.); fanghui@zjut.edu.cn (H.F.); 2 Library, Zhejiang University of Technology, Hangzhou 310014, China

**Keywords:** intimate partner violence, HIV, violence, bibilometric, keywords analysis

## Abstract

Intimate partner violence (IPV) is a worldwide public health problem. Here, a bibliometric analysis is performed to evaluate the publications in the Intimate Partner Violence (IPV) field from 2000 to 2019 based on the Science Citation Index (SCI) Expanded and the Social Sciences Citation Index (SSCI) databases. This work presents a detailed overview of IPV from aspects of types of articles, citations, h-indices, languages, years, journals, institutions, countries, and author keywords. The results show that the USA takes the leading position in this research field, followed by Canada and the U.K. The University of North Carolina has the most publications and Harvard University has the first place in terms of h-index. The London School of Hygiene and Tropical Medicine leads the list of average citations per paper. The *Journal of Interpersonal Violence*, *Journal of Family Violence* and *Violence Against Women* are the top three most productive journals in this field, and Psychology is the most frequently used subject category. Keywords analysis indicates that, in recent years, most research focuses on the research fields of “child abuse”, “pregnancy”, “HIV”, “dating violence”, “gender-based violence” and “adolescents”.

## 1. Introduction

Intimate partner violence (IPV) is a common and worldwide health concern [1,2,3,4,5,6,7,8]. According to the World Health Organization (WHO), IPV includes “any behavior by an intimate partner or ex-partner that causes physical, sexual or psychological harm, including acts of physical aggression, sexual coercion, psychological abuse and controlling behaviors” [9]. According to a WHO report in 2013 [10], over one in three women worldwide have experienced physical and/or sexual partner violence, or sexual violence by a non-partner. IPV levels vary in different regions due to a variety of cultural, economic level, social system, and religious reasons, with the highest prevalence in Africa, the Eastern Mediterranean and the South–East Asia Regions, followed by the Americas. High-income regions, the European and the Western Pacific Regions have a relatively low prevalence [10]. Since IPV is associated with many serious physical and mental health consequences: physical injury [11,12,13,14], post-traumatic stress disorder [15,16,17], HIV infections [18,19,20,21], induced abortion [22,23,24], alcohol use disorders [25,26,27,28,29], adolescent pregnancy [30,31,32,33], dating violence [30,34,35,36,37], and more, scholars from many countries have been participating in the study of IPV and how to prevent the violence [38,39,40,41,42,43,44].

According to the literature, America is the earliest region to study IPV. E.J. Alpert. proposed in 1995 that physicians can play an important role in the early intervention of IPV through querying women who were treated for emergency care [45]. Over the ensuing few years, many strategies were proposed to prevent IPV, such as training programs [46], abuse screening [7,38,40], and reducing poverty and alcohol consumption [41]. Since the WHO released the “World report on violence and health” in 2002, more and more countries joined the IPV research. The collaborations between regions or countries also are increasing.

Recently, bibliometric analysis has been an effective tool to quantitatively analyze academic publications to evaluate the research trends in different research fields, such as health care science services [47,48,49,50,51], Psychology [52,53], Economics [54,55], Energy [11,51,56,57] and Ecology [58,59]. Bibliometrics, first proposed by Alan Pritchard in a paper in 1969, is defined as “the application of mathematics and statistical methods to books and other media of communication” [60]. To our knowledge, this is the first time assessing the IPV research field using bibliometric methods. The aim of this research is to provide a broad overview on the IPV research area, including the following aspects: (1) the main contributors: country, institute, research group; (2) collaboration patterns: cooperation between countries; (3) the most productive journals; (4) top papers with highest citation numbers; (5) research trends by analyzing the author keywords. This study demonstrates the research focuses and hotspots of IPV research, which enable readers to understand the trajectories, key elements on the theoretical and practical contributions, and the future challenges of IPV.

## 2. Methods

The analysis was based on the papers related to “Intimate Partner Violence” which were obtained from the Science Citation Index-Expanded (SCI-E) and Social Sciences Citation Index (SSCI) during the period from 2000 to 2019. The data was retrieved through the “Web of Science Core collection” by searching the title, abstract, author keywords and *KeyWords plus* with the search formula of “Intimate partner violence” or “Intimate partner abuse” or “spous* violence” or “spous* abuse” or “wife violence” or “wife abuse” or “husband violence” or “husband abuse” on 20 July 2020. The data of the top 25 authors in “Intimate Partner Violence” and citation analyses were acquired on 20 July 2020. Keywords and international cooperation were analyzed using the Derwent Data Analyzer (DDA) software. The Impact Factor (IF) for each journal was determined according to the report from the 2019 Journal Citation Reports (JCR). Note that some related publications that do not use the above search formula may not be included in this analysis.

## 3. Results

### 3.1. Number and Types of Publications

**Figure 1:**
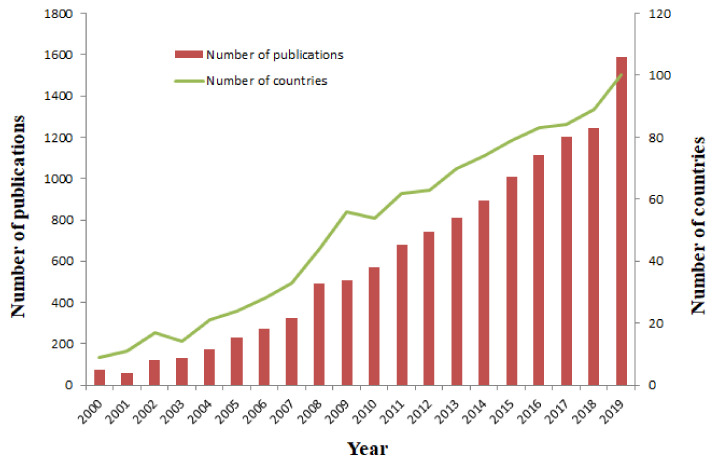
The number of the publication and number of countries by year.

All told, 13,515 papers met the search criteria mentioned above, including 13 article types. They were: articles (11,450), reviews (925), meeting abstracts (550), editorial materials (333), proceedings papers (278), early access (188), letters (94), corrections (41), book reviews (32), book chapters (19), news items (9), reprints (4) and a retracted publication (1). The vast majority of articles and reviews were published in English (12,044; 97.325%), followed by Spanish (180; 1.445%), Portuguese (65; 0.525%), German (32; 0.259%), French (20; 0.162%), Turkish (14; 0.113%), Slovenian (6; 0.048%), Italian (4; 0.032%), Croatian (4; 0.032%), Polish (2; 0.016%), Korean (1; 0.008%), Afrikaans (1; 0.008%) and Hungarian (1; 0.008%). The following analysis was only based on the articles and reviews which were the majority of the publications in this field. Figure 1 shows the annual analysis of published papers and the number of countries. It is clear that the number of annual publications and countries have been increasing at a relatively high rate since 2002. This is attributed possibly to the report from the WHO [9]. Until 2019, 151 countries or regions have participated in IPV research.

The top 30 most productive countries in the IPV research field are shown in Table 1. The USA led the list with the most publications (7947) and highest h-index (149). Canada was in the second position, but the amount of publications is only 12% of that from the USA. Other productive countries include the UK (899), Australia (631), Spain (554), South Africa (513), and Sweden (352). Switzerland took the first position of average citations per paper (71.73). The UK is listed in the second position (29.72), followed by South Africa (29.04), Uganda (24.39), the USA (24.19), India (23.29) and Bangladesh (22.06).

### 3.2. Cooperation of Countries

Shown in Table 1, all the countries from Africa had a very high share of internationally collaborative papers, especially Kenya and Uganda. Ten European countries held a relatively high share of cooperative publications. Especially, Switzerland had an 84.17% share of co-author papers with other countries or regions. It is worth mentioning that, although the USA was the most active country—collaborating with 119 other countries or regions—over 80% of the papers published independently were from the USA. Altogether, most productive countries had frequent cooperation with other countries or regions.

**Figure 2:**
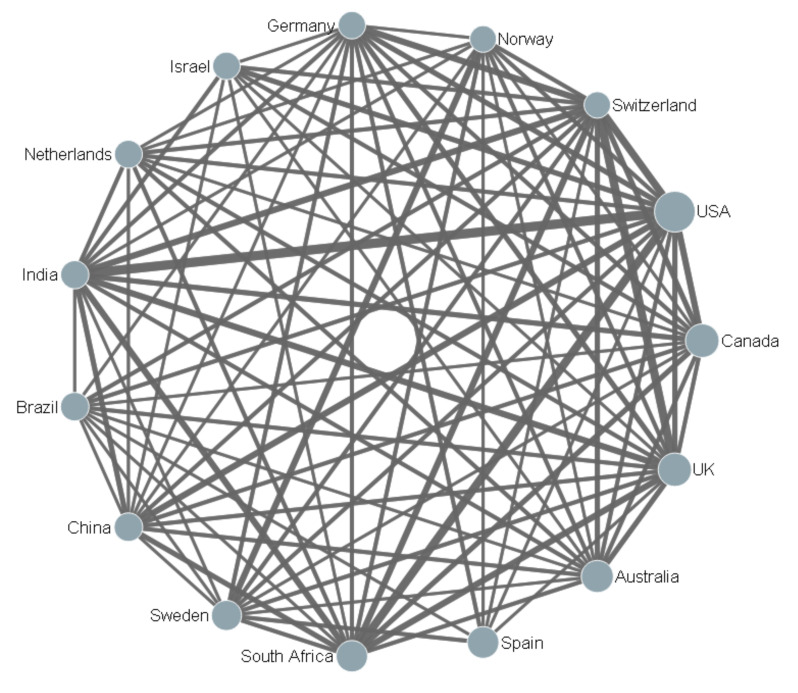
Collaborative relationships among the top 15 most productive countries.

The academic collaboration network of the top 15 most productive countries is shown in Figure 2. Derwent Data Analyzer (DDA) software was applied to draw the network diagram on the basis of a co-occurrence matrix. The size of the nodes is according to the number of publications and the thickness of the connecting lines represent the frequency of cooperation. It is clearly demonstrated that the USA cooperated most frequently with South Africa, India, the UK, and Switzerland with strong collaboration relationships. Furthermore, the USA, the UK, Australia, South Africa, Germany, and Switzerland had the biggest collaboration network within the top 15 most productive countries.

### 3.3. Contribution of Leading Institutes

A total of 6684 institutes have participated in the study of IPV. The top 20 productive institutes, which were from the top four most productive countries, are shown in Table 2. Among them, seventeen institutes are located in the USA, one in the Canada, the UK and Australia respectively, which indicates again that the USA dominates the IPV research area. The University of North Carolina ranks first in terms of total publications, followed by Johns Hopkins University and the University of Michigan. The London School of Hygiene and Tropical Medicine holds the first position for average citations per paper (ACCP). Harvard University has the highest h-index value. It is worth noting that there is no institute from Asia, Africa or Oceania on this list. We expect more countries will increase their funding input and strengthen international and domestic cooperation to prevent IPV.

Additionally, we analyzed the share of cooperative publications between institutes (see Table 2). It can be seen that all 20 of the most productive institutions have very high collaboration rates, especially the University of California-San Diego and Harvard University. It suggests that IPV research requires the cooperation of multiple institutions such as: universities, hospitals, and sectors of government and non-government.

### 3.4. Contribution of Leading Authors

The top 25 most productive authors are shown in Table 3, based on the number of publications. J.C. Campbell led the list with 151 papers followed by J.G. Silverman (94) and G.L. Stuart. (87). Regarding the average citation per paper, C. Watts ranked first with 71.92 followed by R. Caetano (64.27), and R. Jewkes (62.19). The highest h-index was achieved by J.C. Campbell (43). Among these top 25 productive authors, 18 authors were in the USA, two in the UK and Spain, and one in Canada, South Africa, and Australia, respectively.

### 3.5. Contribution of Leading Research Areas and Journals

Twelve thousand three hundred and seventy-five papers related to IPV have been published in about 103 research areas in SCI and SSCI databases, among which the top 20 are listed in Table 4. ‘Psychology’ ranked in the first position in terms of the total publications and h-index, followed by ‘Family Studies’, Public Environmental Occupational Health’ and ‘Criminology Penology’. ‘General Internal Medicine’ led the list of the ACCP (45.38), followed by ‘Neurosciences Neurology’, and ‘Pediatrics’.

**Figure 3:**
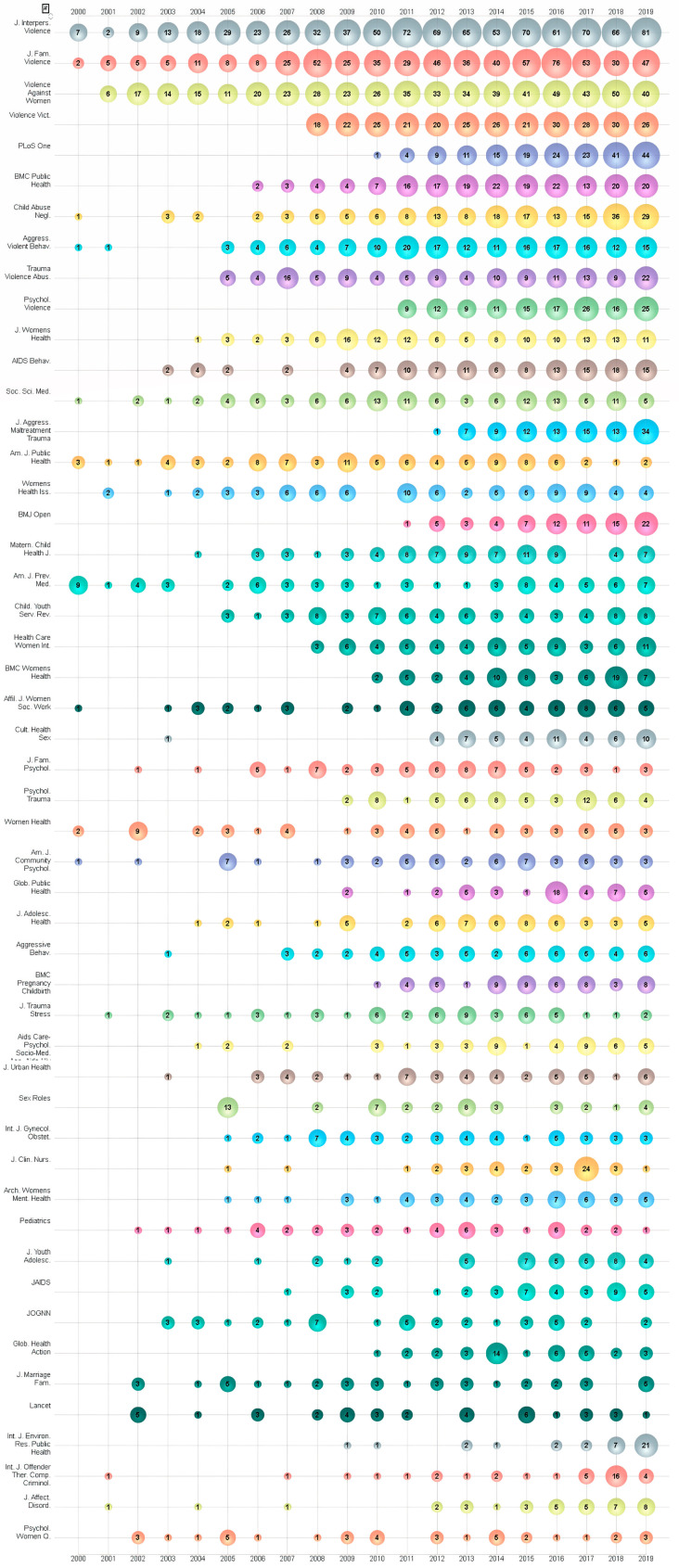
Bubble chart of the Top 50 productive journals by year.

The 12,375 papers related to IPV research during 2000–2019 were published in 1454 journals. The top 50 journals in terms of the number of total publications are shown in Table 5. Approximately 48% of the papers were published in these top 50 productive journals. The top five journals produced 2590 papers with a 21.15% share of the publications. A bubble chart of the top 50 productive journals by year is shown in Figure 3. The *Journal of Interpersonal Violence*, the *Journal of Family Violence* and *Violence Against Women* were the top three most productive journals, with a sharp increase in IPV research outputs during the last decade. It can be clearly seen that there were few articles sparsely distributed in most of the top 50 journals from 2000–2007, however, there has been a rapid growth in publications since 2008. It is clear to see that more and more research workers have contributed to the ‘*International Journal of Environmental Research and Public Health*’. It is worth noting that Lancet (IF = 60.392), one of the most authoritative academic journals in the world medical community and one of the most influential SCI journals, is in the 46th position on the list. This suggests that IPV is a popular and important research area.

### 3.6. An Analysis of Keywords

**Figure 4:**
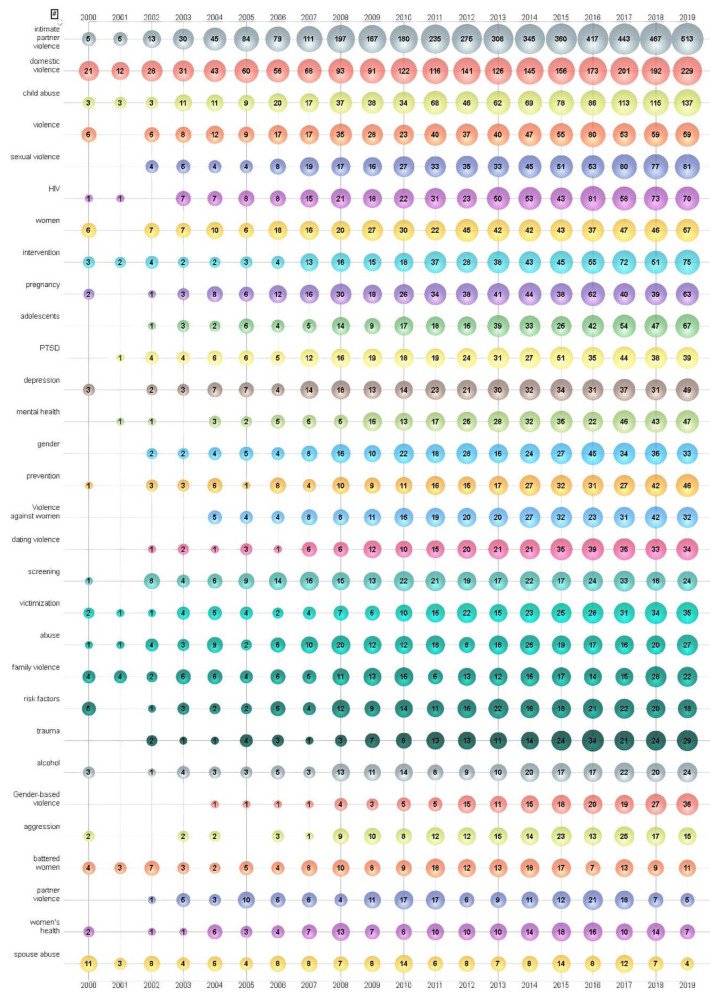
Bubble chart of top 30 author keywords by year.

To elucidate the main focus and research trend of IPV research, 10,085 author keywords from 12,375 papers were analyzed. The raw data were cleaned to ensure that keywords with the same meanings were represented by one unified word. Among the author keywords, 6794 (68%) were used only once and 1279 (13%) used twice. However, the top 30 most used author keywords appeared 16,614 (35%) times. The large number of once-only author keywords may indicate a wide range of interests in IPV research. As a bubble chart can clearly express in 3D values, using the bubble size as the third dimension, one can be applied to track research frontiers [51,61,62,63]. The top 30 author keywords by year are shown in Figure 4. Using visual bubble charts, the development trend of research can be clearly presented. Note that the number on the bubble represents author keyword occurrence frequencies and the number of publications.

### 3.7. An Analysis of the Most Cited Papers

Although the citation impact of a paper depends on many factors [64], it is still a measure of its influence in this research field. The top 20 most highly cited publications are presented in Table 6. The most highly cited paper was “Health consequences of intimate partner violence.” published in the *Lancet* by Campbell, J.C. It led the list of total times cited with 1865 and held the second position for annual citations. “The Epidemiology of Depression Across Cultures” [65], authored by Kessler and Bromet, took the first position for annual citations with 128.00. “Prevalence of intimate partner violence: findings from the WHO multi-country study on women’s health and domestic violence” authored by Garcia–Moreno, C., et al., ranked in the third position with annual citations of 106.46.

Among these top 20 papers, eight were published in Lancet, and one in Psychological Bulletin, the American Journal of Preventive Medicine, the Annual Review of Public Health, Jama-Journal of the American Medical Association, the Clinical Psychology Review, the Archives of Family Medicine, the Annals of the New York Academy of Sciences, Pediatrics, Child Abuse and Neglect, Aggression and Violent Behavior, the Archives of Internal Medicine, and the Bulletin of The World Health Organization, respectively. The USA contributed nine of them, followed by South Africa (4), Switzerland (3) Canada (2), Australia (1) and Ireland (1), and again indicated that the USA was the leading country in this research field. It is worth noting that three papers from South Africa were related to the study of the relationships between IPV and HIV infection and prevention in South Africa. Through analyzing the publications about IPV, we found that 1547 papers were associated with HIV research and a 34% share of the publications was related to Africa. It suggested that more and more scholars agree that HIV and IPV are related to some extent [19,21,66,67,68].

## 4. Discussion

One hundred and fifty-one countries contributed 12,357 publications to Intimate Partner Violence (IPV) research from 2000 to 2019, indicating that IPV is a global public health problem and attracting worldwide attention. It is clear that the number of publications has increased steadily since the WHO released the first World Report on Violence and Health in 2002. The number of papers from 2010–2019 was 9884, which represented 80% of the total number of publications. Also, the number of countries which were involved in IPV research increased every year, except for several fluctuations, which indicates that more and more countries have put their efforts to study and prevent IPV.

North America, Western Europe, and Australia were the most active regions in the research of IPV. This was further confirmed by the most active institutions and authors. There was no institute from Asia and Africa in the top 20 most productive institutions. China and India, as the world’s most populous countries, had very low productivity. One possible reason might be the traditional culture difference, funding input, and economic level. Another possible reason is that while the WoS database is comprehensive, some journals published from India, China, and other Asian and African countries are not indexed in WoS. Furthermore, as most SCI papers are published in English, some non-native English-speaking researchers might not produce high quality papers due to the language problem to some extent. These thoughts might explain the low productivity from Asia and Africa.

The obvious change in the number on the bubble of the author keywords showed the trend of IPV research: “intimate partner violence” (4399 times) was the most frequently used keyword and increased sharply during the last ten years (2008–2019), followed by “domestic violence” (2166 times), “child abuse” (985 times), “violence” (650 times), “sexual violence” (611 times), and “HIV/AIDs” (605 times). It is worth mentioning that, among the top 30 author keywords, five were related to “woman”, including “women”, “pregnancy” “violence against women”, “battered women” and “women’s health” and two were related to adolescents and children, including “adolescents” and “child abuse”, which indicates that the biggest victims of IPV are women and children. Studies on the impact of children and young adolescent’s exposure to IPV have attracted great attention from the scholars over the last two decades [33,34,36,69,70]. This trend reflects on the one-in-four of the total 12,357 papers being related to children. Additionally, “child abuse” “pregnancy”, “HIV”, “dating violence”, “gender-based violence” and “adolescents” were used at a very low frequency during 2000–2007 but increased rapidly during the last decade, which might be the new emerging research direction.

The top 20 cited publications are shown in Table 6. The article with the highest citation was a review article published in 2002 and discussed the increased health problems caused by IPV [14]. Overall, five papers were published in 2002. Therefore, 2002 was a milestone year in IPV research. The article “The world report on violence and health” analyzed and summarized the main points of the first report on violence and health released by the WHO in 2002 [4]. “Prevalence of intimate partner violence: findings from the WHO multi-country study on women’s health and domestic violence” was published in *Lancet* in 2006 and discussed the prevalence of IPV in 10 mainly low and middle-income countries [5]. The report, “Prevalence and health effects of intimate partner violence and non-partner sexual violence”, was released by the WHO in 2013 and demonstrated that 30% of all women from over 80 countries have experienced violence by an intimate partner [10]. The WHO, with other agencies, launched a RESPECT women program to prevent violence against women in 2019 [71].

## 5. Conclusions

Here, we presented a general overview of the Intimate Partner Violence (IPV) research area in terms of leading countries, institutes, and research trends. The USA definitely led IPV research with the most publications and highest h-index. There was no doubt that more and more countries have been participating in IPV research. Since IPV is a world health issue, we expect that, as more and more researchers join this research area, more results will be published based on the collaboration between different research groups all over the world, which will continue to make an effort to stop or prevent IPV. Needless to say, how to prevent IPV more effectively is still a big challenge, although many scholars have made various suggestions to intervene or stop IPV. Furthermore, more and more researchers have recognized that IPV is associated with women’s vulnerability to HIV. We expect more research will focus on these areas.

This study can help potential researchers to quickly understand IPV globally. It also can provide useful information for relevant research in terms of identifying the research trends and potential collaborators, for example. Additionally, this study can help policy makers improve policymaking to prevent IPV.

## Figures and Tables

**Table 1:** Contribution and impact of the top 30 most productive countries in Intimate Partner Violence (IPV) research.

Rank	Country	TP	TC	ACCP	h-Index	SP (%)	nCC	Region
1	USA	7947	196,711	24.75	149	19.51	119	Americas
2	Canada	971	19,666	20.25	65	44.28	72	Americas
3	UK	899	26,718	29.72	71	61.07	90	Europe
4	Australia	631	11,555	18.31	45	46.59	65	Oceania
5	Spain	554	5756	10.39	34	30.69	46	Europe
6	South Africa	513	14,896	29.04	54	71.73	60	Africa
7	Sweden	352	5367	15.25	33	56.25	60	Europe
8	P.R. China	220	3081	14.00	29	50.91	35	Asia
9	Brazil	218	2901	13.31	25	33.94	29	Americas
10	India	200	4658	23.29	37	74.50	30	Asia
11	Netherlands	181	2970	16.41	26	48.62	35	Europe
12	Israel	166	2402	14.47	25	35.54	23	Asia
13	Germany	159	2431	15.29	23	65.41	45	Europe
14	Norway	127	2136	16.82	23	55.12	37	Europe
15	Switzerland	120	8608	71.73	36	84.17	47	Europe
16	Italy	117	1460	12.48	19	61.54	43	Europe
17	New Zealand	104	2017	19.34	24	36.54	19	Oceania
18	Uganda	103	2512	24.39	25	95.14	26	Africa
19	Turkey	102	809	7.93	15	17.65	8	Asia
20	Kenya	96	1536	16.00	20	96.88	29	Africa
21	Mexico	95	1188	12.51	19	69.47	19	Americas
22	South Korea	87	687	7.90	14	56.32	9	Asia
23	Bangladesh	85	1875	22.06	21	81.18	17	Asia
24	Japan	79	899	11.38	15	48.10	18	Asia
25	Nigeria	75	1115	14.87	19	58.67	30	Africa
26	Tanzania	70	858	12.26	18	92.86	28	Africa
27	Portugal	67	525	7.84	12	46.27	21	Europe
28	Ethiopia	64	861	13.45	17	65.08	25	Africa
29	Belgium	56	949	16.95	16	62.50	35	Europe
30	Chile	52	524	10.08	13	82.69	20	Americas

Note: TP total paper, TC total citations, ACCP average citations per paper, SP Share of publications. nCC number of cooperative countries or regions.

**Table 2:** The Top 20 most productive institutions for publications, citations, and h-indices during 2000–2019.

Institutions	TP	TC	ACCP	h-Index	SP (%)	Country
University of North Carolina	348	11,970	34.40	55	75.29	USA
Johns Hopkins University	337	13,427	39.84	59	89.61	USA
University of Michigan	295	6361	21.41	37	81.02	USA
Columbia University	282	7158	25.38	45	90.07	USA
Boston University	281	9225	32.83	52	93.95	USA
Emory University	280	7420	26.50	40	82.86	USA
Harvard University	260	13,425	51.63	60	98.85	USA
University of Washington	250	8112	32.45	48	84.40	USA
University of California San Francisco	228	5567	24.42	38	90.79	USA
University of Toronto	223	4882	21.89	35	90.58	Canada
Yale University	215	4146	19.28	34	85.58	USA
Johns Hopkins Bloomberg School of Public Health	192	4084	21.27	34	91.67	USA
London School of Hygiene & Tropical Medicine	190	10,575	55.66	45	92.11	UK
Michigan State University	188	3574	19.01	31	65.96	USA
University of Pittsburgh	174	3868	22.23	36	87.93	USA
Ctr Dis Control & Prevent	171	6772	39.60	35	69.01	USA
University of California San Diego	171	4643	27.15	36	98.83	USA
University of Pennsylvania	168	3553	21.15	32	86.31	USA
University of Illinois	161	3241	20.13	32	76.40	USA
University of Melbourne	157	2889	18.40	28	87.90	Australia

Note: TP total paper, TPR% the percentage of articles or journals in total publications, TC total citations, ACCP average citations per paper, SP Share of publications.

**Table 3:** Contribution of the top 25 authors in IPV research.

Rank	Author	TP	TPR%	TC	ACCP	h-Index	Institute (Current), Country
1	Campbell, J.C.	151	1.233	8310	55.03	41	Johns Hopkins University, USA
2	Silverman, J.G.	94	0.768	4983	53.01	36	Harvard University, USA
3	Stuart, G.L.	87	0.710	2615	30.06	28	The University of Tennessee, USA
4	Decker, M.R.	86	0.702	3751	43.62	35	Johns Hopkins Bloomberg School of Public Health, USA
5	Jewkes, R.	79	0.645	4913	62.19	30	South African Medical Research Council, South Africa
6	Raj, A.	78	0.637	3978	51.00	32	University of California San Diego, USA
7	Miller, E.	71	0.580	2037	28.69	25	University of Pittsburgh, USA
8	Vives-Cases, C.	64	0.523	728	11.38	14	University of Alicante, Spain
9	Shorey, R. C.	62	0.506	1261	20.34	17	Ohio University, USA
10	O’Campo, R.	61	0.498	2665	42.98	26	St Michaels Hospital, Toronto, Canada
11	Watts, C.	60	0.490	4315	71.92	30	London School of Hygiene & Tropical Medicine, UK
12	Caetano, R.	59	0.482	3792	64.27	36	Pacific Institute for Research and Evaluation, USA
13	Feder, G	56	0.457	1862	33.25	20	University of Bristol, UK
14	Graham-Bermann, S.A.	55	0.449	1055	19.18	18	University of Michigan, USA
15	Yount, K.M.	55	0.449	843	15.33	19	Emory University, USA
16	Cerulli, C.	54	0.441	904	16.74	15	University of Rochester, USA
17	El-Bassel, N.	54	0.441	1484	27.48	21	Columbia University, USA
18	Lila, M.	53	0.433	737	13.91	17	University of Valencia, Spain
19	McFarlane, J.	53	0.433	862	16.26	13	Texas Woman’s University, USA
20	Glass, N.	52	0.425	1463	28.13	17	Johns Hopkins, USA
21	Hegarty, K	52	0.425	1126	21.65	16	University of Melbourne, Australia
22	Stephenson, R	51	0.416	1144	22.43	17	University of Michigan, USA
23	Kaslow, N.J.	50	0.408	1391	27.82	21	Emory University, USA
24	Gilbert, L	48	0.392	1450	30.21	20	Columbia University, USA
25	Sullivan, T.P.	47	0.384	687	14.62	14	Yale University, USA

Note: TP total paper, TPR% the percentage of articles of journals in total publications, TC total citations, ACCP average citations per paper.

**Table 4:** Contribution of the Top 20 research areas in IPV research.

Research Areas	TP	TC	ACCP	h-Index
Psychology	4262	83,592	19.61	105
Family Studies	2670	50,493	18.91	87
Public Environmental Occupational Health	2608	61,018	23.40	102
Criminology Penology	2253	40,510	17.98	81
Women’s Studies	1182	21,812	18.45	62
Psychiatry	1159	22,239	19.19	66
Social Work	911	17,614	19.33	65
General Internal Medicine	837	37,983	45.38	87
Nursing	581	6289	10.82	35
Obstetrics Gynecology	569	12,284	21.59	52
Biomedical Social Sciences	463	12,269	26.50	58
Substance Abuse	365	8983	24.61	50
Health Care Sciences Services	361	5521	15.29	37
Pediatrics	268	7243	27.03	46
Social Sciences Other Topics	220	2897	13.17	27
Science Technology Other Topics	219	3673	16.77	32
Infectious Diseases	198	5109	25.80	39
Neurosciences Neurology	194	6317	32.56	42
Sociology	186	3459	18.60	33
Government Law	180	1914	10.63	23

Note: TP total paper, TPR% the percentage of share publications, TC total citations, ACCP average citations per paper.

**Table 5:** Contribution of the top 50 most productive Journals in IPV research.

No.	Journal Name	IF2019	No.	Journal Name	IF2019
1	Journal of Interpersonal Violence	3.573	26	Psychological Trauma Theory Research Practice and Policy	2.595
2	Journal of Family Violence	1.357	27	Women’s Health	1.095
3	Violence against Women	1.797	28	American Journal of Community Psychology	1.509
4	Violence and Victims	0.598	29	Global Public Health	1.791
5	Plos One	2.740	30	Journal of Adolescent Health	3.900
6	BMC Public Health	2.521	31	Aggressive Behavior	2.219
7	Child Abuse Neglect	2.569	32	BMC Pregnancy and Childbirth	2.239
8	Aggression and Violent Behavior	2.893	33	Journal of Traumatic Stress	1.926
9	Trauma Violence Abuse	6.325	34	Aids Care-Psychological and Socio-Medical Aspects of Aids/HIV	1.894
10	Psychology of Violence	2.381	35	Journal of Urban Health Bulletin of The New York Academy of Medicine	2.356
11	Journal of Women’s Health	1.933	36	Sex Roles	2.409
12	Aids and Behavior	3.147	37	International Journal of Gynecology Obstetrics	2.216
13	Social Science Medicine	3.616	38	Journal of Clinical Nursing	1.972
14	Journal of Aggression Maltreatment Trauma	1.030	39	Archives of Women’s Mental Health	2.500
15	American Journal of Public Health	6.464	40	Pediatrics	5.359
16	Women’s Health Issues	2.355	41	Journal of Youth and Adolescence	3.121
17	BMJ Open	2.496	42	Jaids-Journal of Acquired Immune Deficiency Syndromes	3.475
18	Maternal and Child Health Journal	1.890	43	Jognn Journal of Obstetric Gynecologic and Neonatal Nursing	1.250
19	American Journal of Preventive Medicine	4.420	44	Global Health Action	2.162
20	Children and Youth Services Review	1.521	45	Journal of Marriage and Family	2.215
21	Health Care for Women International	0.970	46	Lancet	60.392
22	BMC Women’s Health	1.544	47	International Journal of Environmental Research and Public Health	2.849
23	Affilia Journal of Women and Social Work	1.085	48	International Journal of Offender Therapy and Comparative Criminology	1.026
24	Culture Health Sexuality	1.969	49	Journal of Affective Disorders	3.892
25	Journal of Family Psychology	1.840	50	Psychology of Women Quarterly	2.444

Note: IF: impact factor.

**Table 6:** The Top 20 most cited publications in IPV research field during 2000–2019.

No	Authors	Title	Total Citation	Citation/Year	Journal/IF2019	Publication Year	Country (Reprint Address)
1	Campbell, J.C.	Health consequences of intimate partner violence.	1865	109.71	Lancet/60.392	2002	USA
2	Garcia-Moreno, C.; Jansen, H.A.F.M.; Ellsberg, M.; et al.	Prevalence of intimate partner violence: Findings from the WHO multi-country study on women’s health and domestic violence.	1384	106.46	Lancet/60.392	2006	Switzerland
3	Archer, J.	Sex differences in aggression between heterosexual partners: A meta-analytic review.	1350	71.05	Psychological Bulletin/20.850	2007	Canada
4	Coker, A.L.; Davis, K.E.; Arias, I.	Physical and mental health effects of intimate partner violence for men and women.	1169	68.76	American Journal of Preventive Medicine/4.420	2002	USA
5	Krug, E.G.; Mercy, J.A.; Dahlberg, L.L.; et al.	The world report on violence and health.	948	55.76	Lancet/60.392	2002	Switzerland
6	Ellsberg, M.; Jansen, H.A.F.M.; Heise, L.; et al.	Intimate partner violence and women’s physical and mental health in the WHO multi-country study on women’s health and domestic violence: An observational study.	891	81.00	Lancet/60.392	2008	Switzerland
7	Jewkes, R.	Intimate partner violence: Causes and prevention.	814	47.88	Lancet/60.392	2002	South Africa
8	Dunkle, K.L; Jewkes, R.K.; Brown, H.C.; et al.	Gender-based violence, relationship power, and risk of HIV infection in women attending antenatal clinics in South Africa.	797	53.13	Lancet/60.392	2004	South Africa
9	Kessler, R.C., Bromet, E.J.	The Epidemiology of Depression Across Cultures	768	128.00	Annual Review of Public Health/16.463	2013	USA
10	Silverman, J. G., Raj, A., Mucci, L. A.; et al.	Dating violence against adolescent girls and associated substance use, unhealthy weight control, sexual risk behavior, pregnancy, and suicidality.	735	40.83	Jama-Journal of the American Medical Association/45.540	2001	USA
11	Babcock, J.C.; Green, C.E.; Robie, C.	Does batterers’ treatment work? A meta-analytic review of domestic violence treatment	685	45.67	Clinical Psychology Review/10.225	2004	USA
12	Jewkes, R.K.; Dunkle, K.; Nduna, M., et al.	Intimate partner violence, relationship power inequity, and incidence of HIV infection in young women in South Africa: A cohort study.	659	73.22	Lancet/60.392	2010	South Africa
13	Shin, L.M.; Rauch, S.L.; Pitman, R.K.	Amygdala, medial prefrontal cortex, and hippocampal function in PTSD	614	43.86	Annals of the New York Academy of Sciences/4.728	2005	USA
14	Coker, A.L.; Smith, P.H, Bethea, L.; et al.	Physical health consequences of physical and psychological intimate partner violence.	611	32.16	Archives of Family Medicine/2.878 (Year 2002)	2000	USA
15	Tremblay, R.E.; Nagin, D.S.; Seguin, J.R.; et al.	Physical aggression during early childhood: Trajectories and predictors	570	38.00	Pediatrics/5.359	2004	Canada
16	Holt, S., Buckley, H., Whelan, S.	The impact of exposure to domestic violence on children and young people: A review of the literature.	561	51.00	Child Abuse & Neglect/2.569	2008	Ireland
17	Stith, S.M.; Smith, D.B.; Penn, C.E.	Intimate partner physical abuse perpetration and victimization risk factors: A meta-analytic review	556	37.07	Aggression and Violent Behavior/2.893	2004	USA
18	Campbell, J.; Jones, A.S.; Dienemann, J.; et al.	Intimate partner violence and physical health consequences.	536	31.53	Archives of Internal Medicine/17.333 (Year 2014)	2002	USA
19	Fisher, J.; de Mello, M.C.; Patel, V.; et al.	Prevalence and determinants of common perinatal mental disorders in women in low- and lower-middle-income countries: A systematic review.	526	75.14	Bulletin of The World Health Organization/6.960	2012	Australia
20	Pronyk, P.M.; Hargreaves, J.R.; Kim, J.C.; et al.	Effect of a structural intervention for the prevention of intimate-partner violence and HIV in rural South Africa: a cluster randomised trial.	521	40.08	Lancet/60.392	2006	South Africa

Note: Total Citation/Year: Total Citation/ (2019-Publication Year).
